# “Pre-Treatment“ and “Post-Treatment” Systemic Inflammatory Markers: Is There Any Prognostic Role for Metastatic Cervical Cancer on Bevacizumab Containing Treatment?

**DOI:** 10.3390/medicina61061100

**Published:** 2025-06-17

**Authors:** Serkan Yaşar, Ahmet Kadıoğlu, Arif Akyildiz, Nadiye Sever, Mehmet Emin Büyükbayram, Mehmet Bilici, Elanur Karaman, Mehmet Uzun, Murat Bardakcı, Caglar Koseoglu, Irem Bilgetekin, Mehmet Cihan İçli, Alper Türkel, Zafer Arık, Murat Sarı, Tugba Yavuzsen, Mehmet Ali Nahit Sendur, İsmail Erturk, Mutlu Dogan

**Affiliations:** 1 Department of Medical Oncology, Dr Abdurrahman Yurtaslan Ankara Oncology Training & Research Hospital, University of Health Sciences, 06200 Ankara, Türkiye; 2 Department of Medical Oncology, Hacettepe University Cancer Institute, 06230 Ankara, Türkiye; 3 Department of Medical Oncology, Faculty of Medicine, Marmara University, 34854 Istanbul, Türkiyedrmuratsari@gmail.com (M.S.); 4 Department of Medical Oncology, Faculty of Medicine, Atatürk University, 25240 Erzurum, Türkiye; 5 Department of Medical Oncology, Faculty of Medicine, Karadeniz Technical University, 61080 Trabzon, Türkiye; 6 Department of Medical Oncology, Institute of Oncology, Dokuz Eylul University, 35340 İzmir, Türkiye; 7 Department of Medical Oncology, Ankara Bilkent City Hospital, 06800 Ankara, Türkiye; 8 Department of Medical Oncology, Gulhane Training and Research Hospital, University of Health Sciences, 06200 Ankara, Türkiye; 9 Department of Medical Oncology, Memorial Ankara Hospital, 06520 Ankara, Türkiye

**Keywords:** cervix, bevacizumab, systemic inflammatory markers

## Abstract

*Background and Objectives*: Despite developments in cervical cancer (CC) treatment, an advanced stage is a poor prognostic factor. Cervical cancer is an immunogenic tumor in which viruses, like HPV, play a role in carcinogenesis. Therefore, systemic inflammatory markers (SIMs) may have prognostic value. Most studies on SIMs focus on the early stage by evaluating pretreatment levels. This study aims to evaluate the prognostic and predictive values of both pretreatment and post-treatment parameters at the advanced stage, as well as treatment efficacy after progression with first-line treatment. *Materials and Methods*: A total of 133 advanced-stage CC patients with progression on first-line platin–paclitaxel and bevacizumab were evaluated retrospectively. Demographic and histopathological characteristics were recorded along with treatment details. Pre-treatment baseline blood parameters and post-treatment follow-up values were recorded to calculate SIMs as the neutrophil-to-lymphocyte ratio (NLR), platelet-to-lymphocyte ratio (PLR), systemic immune-inflammation index (SII), and systemic inflammatory response index (SIRI). *Results*: Median values for SIMs were accepted as cut-off values. Post-treatment values demonstrated stronger predictive power, with pre-treatment SIRI and NLR being significant only in univariate analysis, but not in multivariate analysis. High post-treatment SIRI (>2.1) was correlated with shorter overall survival (OS) and considered a poor prognostic factor. High post-treatment SIRI (>2.1), -SII (>746), and -PLR (>197) emerged as independent prognostic factors for progression-free survival (PFS). Their prognostic values were clearer in the whole population and the metachronous metastatic subgroup. Rechallenge of platinum-based chemotherapy was an option for those who had at least 6 months of PFS with first-line platinum-based chemotherapy. Bevacizumab addition to single-agent or combination regimens led to improved ORR as well. *Conclusions*: Post-treatment SIRI is a promising prognostic factor for OS, while post-treatment SIRI, SII, and PLR may serve as convenient SIMs for PFS. Platinum-based combination chemotherapy reinduction is a feasible second-line treatment strategy, especially with the addition of bevacizumab.

## 1. Introduction

Cervical cancer remains one of the leading causes of cancer-related death among women, particularly in low- and middle-income countries. Despite advances in screening and vaccination for human papillomavirus (HPV), a common cause of cervical cancer, a significant number of patients still present at an advanced stage. [1] Although non-metastatic patients can be treated curatively with surgery, chemotherapy, and/or chemoradiotherapy, the likelihood of cure in metastatic ones is quite low, particularly for those who experience early progression on platinum-based therapy. They have a poor prognosis with a limited number of curative treatment options [2]. Five-year overall survival (OS) rate ranges from 50% to 80% for patients with locally advanced-stage disease. However, the development of metastases leads to a substantial decrease in survival, reducing this rate to as low as 17% [3,4]. Platin–taxane–bevacizumab combination is a standard of care as a first-line setting in metastatic patients. Final analysis of the GOG 240 trial showed a survival benefit with the addition of bevacizumab to the first-line cisplatin–paclitaxel combination regimen [2,3]. Additionally, the Japanese Clinical Oncology Group 0505 trial showed noninferiority of carboplatin (area under the curve, 5 mg per minute) in this combination [5]. Treatment outcomes are often unsatisfying for patients with disease progression on first-line systemic treatment. Non-platinum treatment options, as single agents or combination regimens, are often used as subsequent lines. Median progression-free survival (PFS) is around 3 to 6 months beyond first line, and objective response rates (ORRs) range from 0% to 14%, as well [6,7,8]. Lower ORRs with a short duration of response were reported for single agents, such as pemetrexed, irinotecan, topotecan, ifosfamide, vinorelbine, and gemcitabine as second-line treatment options [6].

Rudolf Virchow first described the relationship between tumor development and chronic inflammation in 1863 [9]. This invention led to an acceleration in this area. The role of inflammation in driving cancer development, metastatic spread, and resistance to treatment is well-established. The tumor microenvironment, shaped by stromal inflammatory cells, actively supports tumor growth, survival, and tumor spread [10,11]. Studies have demonstrated that blood cells, as crucial components of the immune system, play a pivotal role in this process [11]. The prognostic significance of combined inflammation biomarkers and systemic inflammatory markers (SIMs), such as neutrophil-to-lymphocyte ratio (NLR), platelet-to-lymphocyte ratio (PLR), systemic immune-inflammation index (SII), and systemic inflammatory response index (SIRI), has been evaluated in various solid tumor types and hematological malignancies [12,13,14,15]. Most of these studies evaluated their prognostic roles at baseline pre-treatment blood samples of the patients, particularly at the early stage [16,17,18]. However, a limited number of studies have highlighted the prognostic significance of “post-treatment” inflammatory parameters in predicting outcomes and guiding therapeutic strategies [14,15].

A poor prognosis in cervical cancer motivated a search for possible prognostic factors at an advanced stage. Most of the prior studies on cervical cancer have focused on “pre-treatment” values at the early stage [16,17,18]. Therefore, we aimed to evaluate the prognostic value of both “pre-treatment” and “post-treatment” SIMs in metastatic cervical cancer patients on the first-line platinum–taxane–bevacizumab combination regimen. We also aimed to evaluate the efficacy of subsequent treatment outcomes after progression on first-line platin–taxane–bevacizumab treatment. We consider that the evaluation of these points may contribute to the evidence base for the management of this challenging process. 

## 2. Materials and Methods

### 2.1. Patients’ Characteristics

Our study is a multicenter, retrospective observational study, including patients with histopathologically diagnosed HPV-positive cervical cancer in nine experienced cancer centers. The study population consisted of patients with systemic relapse following definitive treatment for locally advanced stage (i.e., metachronous metastasis) or diagnosed with metastatic disease (i.e., “de novo” metastasis). Between 2010 and 2023, a cohort of 133 patients was included, all of whom received first-line platinum–taxane–bevacizumab combination therapy for advanced-stage disease. Patients aged over 18 years with an ECOG performance status of 0–1 were included in the study.

### 2.2. Data Collection

Clinical and pathological features, including demographic characteristics, stage, histopathology, and treatment outcomes, were obtained from hospital registration database. Systemic inflammatory markers were derived from blood-based parameters measured before (i.e., “pre-treatment”) and after (i.e., “post-treatment”) first-line platinum–taxane–bevacizumab treatment. Patients with comorbid conditions, such as acute infections, hematological disorders, secondary malignancies, or rheumatologic diseases, were excluded from the study. Blood parameters within four weeks prior to treatment and at least 4 to 6 weeks following treatment were selected for analysis, with the timing guided by the peak inflammatory response kinetics and average lifespans of cells such as neutrophils, lymphocytes, monocytes, and platelets.

Systemic inflammatory markers, such as the neutrophil-to-lymphocyte ratio (NLR, defined as neutrophil/lymphocyte counts), platelet-to-lymphocyte ratio (PLR, defined as platelet/lymphocyte counts), systemic immune-inflammation index (SII, defined as platelet × neutrophil/lymphocyte counts), and systemic inflammatory response index (SIRI, defined as monocyte × neutrophil/lymphocyte counts), were calculated based on pre- and post-treatment values. The patients were evaluated in subgroups based on both pre-treatment and post-treatment median levels as “cut-off” levels for definition of “low” and “high” levels. Treatment responses were evaluated according to RECIST 1.1 criteria. Objective response rate (ORR) was defined as the sum of complete response (CR) and partial response (PR), while the disease control rate (DCR) was defined as the sum of CR, PR, and stable disease (SD).

All patients included in the study received first-line systemic therapy consisting of paclitaxel (175 mg/m^2^, day 1), cisplatin (50 mg/m^2^, day 1), and bevacizumab (15 mg/kg, day 1), administered every 21 days. Patients who were not eligible for cisplatin received carboplatin (AUC 5, day 1) instead, also on a 21-day cycle. In cases of disease progression following first-line treatment, second-line regimens were initiated, which primarily included gemcitabine, topotecan, docetaxel, or other platinum-based agents.

Bevacizumab and/or immunotherapy were also added to second-line chemotherapy if PFS was at least 6 months with first-line bevacizumab-containing treatment. Immunotherapy could have been given to the patients who supplied it by themselves, since it is not reimbursed in this setting in our country. A total of 92 (69.2%) patients had locally advanced-stage disease, and 43 (46.7%) of them had surgery. Among these patients, 23 (53.4%) had adjuvant chemoradiotherapy. Fifty (54.3%) patients with locally advanced-stage cervical cancer had definitive chemoradiotherapy, as well. Two patients had only radiotherapy.

### 2.3. Statistical Analysis

Survival outcomes were analyzed by using the Kaplan–Meier method, and differences between groups were compared by using the log-rank test. Overall survival was defined as the duration from the initiation of treatment to the date of last follow-up assessment and/or death. Progression-free survival was defined as the duration from treatment initiation to the first progression and/or death. Univariate and multivariate Cox proportional hazards regression analyses were conducted for further evaluation of potential biomarkers for their prognostic significance.

The parameters with a *p*-value < 0.05 in univariate analysis were included in the multivariate model to identify independent prognostic factors for OS and PFS. Hazard ratios (HR) with 95% confidence intervals (CI) were reported. Two-sided *p*-values < 0.05 were considered statistically significant and indicated significant differences in all other analyses. The statistical analysis was conducted using SPSS, version 25.0 (IBM Inc., Armonk, NY, USA).

### 2.4. Results

Median follow-up duration was 25.4 months (IQR: 15.4–44.6). A total of 133 patients diagnosed with locally advanced or metastatic cervical cancer were included in the study. The patients’ characteristics are summarized in Table 1. Median age was 51 years (range: 24–75). A total of 105 (78.9%) patients had squamous cell carcinoma (SCC), and 28 (21.1%) cases had adenocarcinoma histopathology. At diagnosis, 92 (69.2%) patients had locally advanced-stage disease, while 41 (30.8%) patients had “de novo” metastatic disease. We subgrouped the patients according to the FIGO staging system. The patients with FIGO stage (2–4) were included in the study. A total of 48 (36.1%) patients were classified as stage 2, where 42 (31.6%) patients were staged as stage 3, and remaining 43 (32.3%) patients were classified as stage 4, respectively.

“Pre-treatment” median values for NLR, PLR, SII, and SIRI were 3.56, 224, 909, and 2.4. The corresponding “post-treatment” values were 3.1, 197, 746, and 2.1, as well. The prognostic significance of clinicopathological features and SIMs (i.e., NLR, PR, SII, and SIRI) was evaluated in this study, as mentioned above.

### 2.5. Survival Analysis

All of the patients had advanced-stage disease at analysis. The patients were grouped as “de novo” metastatic (n: 41, 30.8%) and metachronous metastatic (n: 92, 69.2%) subgroups for survival analysis. A total of 92 (69.2%) patients had a locally advanced stage at diagnosis and progressed during follow-up after definitive treatment.

Median OS was 34.6 months (95% CI: 21–48.2) for overall population. The patients with “de novo” metastasis had significantly shorter OS. Median OS was 21.1 months (95% CI: 14.6–27.8) for “de novo” metastatic patients, whereas it was 49.3 months (95% CI: 33.1–65.4) for the others with metachronous metastasis, as well (*p* < 0.001).

Survival analysis (i.e., OS and PFS) according to SIMs is detailed below. Survival outcomes based on pre-NLR and post-NLR revealed a significant OS difference in the overall population and the metachronous metastatic subgroup among patients with low pre-NLR (<3.56) (*p* = 0.035, *p* = 0.012) or post-NLR (<3.1) (*p* < 0.001, *p* < 0.001). Median OS was notably longer in patients with low pre-NLR (53.2 vs. 26.2 months for overall population and 63.9 vs. 29.2 months for the metachronous metastatic subgroup). A similar trend was observed for post-NLR, with median OS of 81.5 vs. 25.4 months in the overall population and 85.3 vs. 26.2 months in the metachronous metastatic subgroup. However, no significant OS difference was detected in de novo metastatic patients (*p* = 0.086).

Post-treatment PLR (post-PLR), rather than pre-treatment PLR (pre-PLR), had prognostic significance for all patients and metachronous metastatic ones. There was no statistically significant difference in any group when comparing high and low pre-PLR values (*p*-values for overall, de novo metastatic, and metachronous metastatic groups are as follows: 0.898, 0.768, and 0.901); however, statistically significant differences were observed in post-PLR analyses. Median OS was 62.3 mos vs. 25.4 mos (*p* = 0.006) and 63.9 vs. 26.7 mos (*p* = 0.01), in favor of low post-PLR (<197) in all population and metachronous metastatic subgroups. None of these parameters differed for survival in “de novo” metastatic patients (*p* = 0.073). Similarly, post-treatment SII (post-SII), but not pre-treatment SII (pre-SII), was shown to have prognostic significance in both overall population and metachronous metastatic subgroup, in parallel to the prognostic role of PLR (i.e., insignificance for pre-SII, significance for post-SII). The patients with low post-SII (<746) had significantly longer OS. Median OS was 63.9 mos vs. 25.4 mos in all populations, while it was 81 mos versus 26.7 mos in metachronous subgroup (*p* = 0.001 for both).

**Figure 1:**
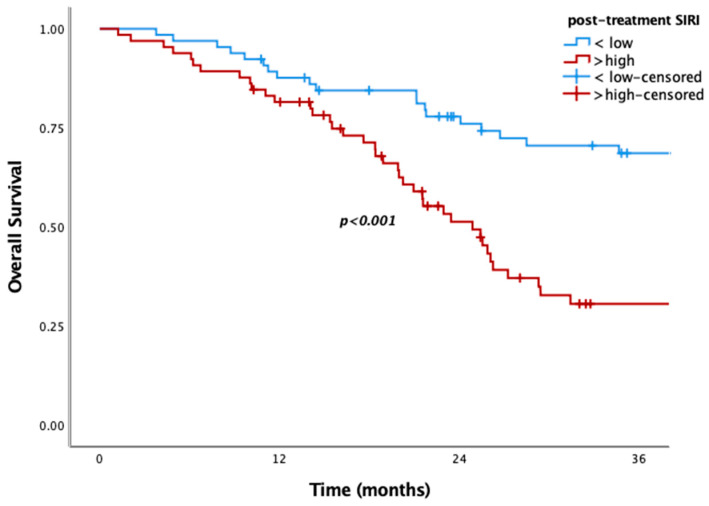
Kaplan–Meier curves according to high and low post-SIRI for overall population.

**Figure 2:**
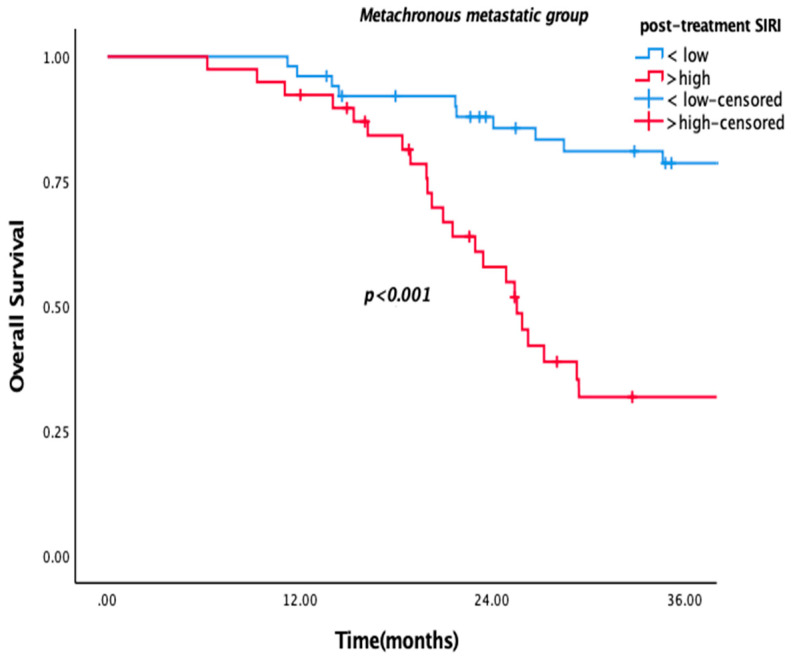
Kaplan–Meier curves according to high and low post-SIRI for metachronous group.

**Figure 3:**
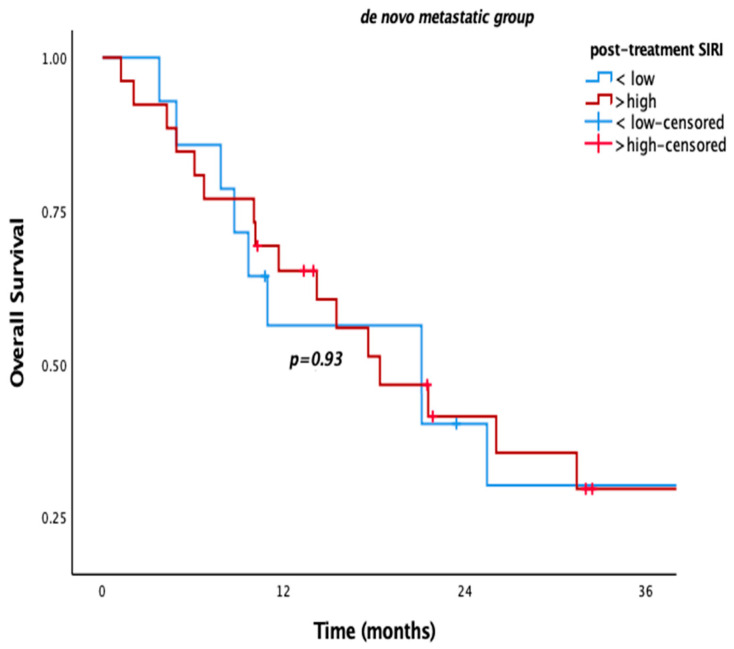
Kaplan–Meier curves according to high and low post-SIRI for de novo metastatic group.

Patients with low pre-SIRI (<2.4) or post-SIRI (<2.1) had significantly better OS in the overall population and the metachronous metastatic subgroup. Median OS was 55.7 vs. 23.4 months in the overall population, while it was 62.3 vs. 26.2 months in the metachronous subgroup for low pre-SIRI (*p* = 0.006, *p* = 0.005, respectively) (Figure 1). Similarly, low post-SIRI was associated with improved survival (85.4 vs. 24.8 months in overall population and 89.4 vs. 25 months in the metachronous subgroup; *p* < 0.001 for both) (Figure 2). However, as with other SIMs, no significant OS difference was observed in de novo metastatic patients (*p* = 0.780, *p* = 0.93) (Figure 3).

The variables included in univariate and multivariate analyses to assess their prognostic significance for OS and PFS were histopathological subtype, age (<65 and ≥65), and pre-/post-SIMs (SII, PLR, NLR, and SIRI). High pre-NLR and SIRI were poor prognostic factors for OS in univariate analysis (Table 2). However, none of them had prognostic significance in multivariate analysis. In post-treatment analyses, SIRI, SII, NLR, and PLR were significant, whereas in the multivariate analysis, only SIRI remained statistically significant (Table 3). So, only post-SIRI was an independent prognostic factor for OS (HR = 2.11, 95% CI: 1.156–3.85, *p* = 0.015).

There was no statistically significant difference for any “pretreatment” parameters for PFS in univariate analysis. Therefore, we could not have performed multivariate analysis using any of them. For post-treatment parameters, post-SIRI, -NLR, -SII, and -PLR were significant in univariate analysis. When they were put into multivariate analysis, post-SIRI, -SII, and -PLR were statistically significant, and each was considered an independent risk factor for PFS (Table 4).

### 2.6. Treatment Outcomes

Of 133 patients, 69.2% had locally advanced-stage disease at diagnosis and progressed during follow-up. None of them had locoregional treatment (i.e., surgery, radiotherapy) at progression after locoregional treatment at diagnosis. Median treatment free interval after definitive treatment was 14 months (IQR: 9–25.73). In overall population, median PFS with first-line “platinum-taxane-bevacizumab” regimen was 8 months (95% CI: 6.54–4.45). No significant difference for response to first-line treatment was observed for either metachronous or “de novo” metastatic subgroups. Median PFS was 9 months vs. 7 months, respectively (*p* = 0.058). Median PFS with second-line treatment was 4 months (95% CI: 3.1–4.8). Due to the variability in second-line treatment options, treatment outcomes of each chemotherapy regimen were assessed separately (Table 5).

Based on the physicians’ choice, the patients received second-line treatments, such as topotecan, gemcitabine, docetaxel, platinum/taxane (only for the patients with a PFS1 of at least 6 months), pembrolizumab, and other single-agent or combination regimens. In certain cases, bevacizumab was added to the treatment as a rechallenge strategy.

In the second-line setting, physicians frequently preferred single-agent gemcitabine therapy (25%), achieving a disease control rate (DCR) of 24%. The addition of platinum to gemcitabine increased the response rate to 33.3%, while triplet therapy with bevacizumab further improved it up to 50%. Following gemcitabine-based therapies, platinum–taxane and bevacizumab rechallenge therapy (17.8%) was the most preferred regimen in our study. It also appeared to be an effective second-line option. Specifically, cisplatin–paclitaxel plus bevacizumab achieved an objective response rate (ORR) of 16.6% and a DCR of 33.3%. Similarly, in patients in whom cisplatin was replaced with carboplatin, the ORR was reported as 11.1% and DCR as 22.2%. Apart from these treatment options, disease control was achieved at varying rates with other single-agent or combination therapies, without any objective response (Table 5). Notably, among three patients receiving pembrolizumab, two demonstrated disease control for over one year.

## 3. Discussion

Despite advancements in screening programs and treatments, cervical cancer remains a major contributor to cancer-related mortality among women [1]. Most of the patients are diagnosed at the nonmetastatic stage; however, metastasis occurs in 15–61% of patients within the first two years [19,20]. Studies evaluating the efficacy of second-line treatment options for advanced-stage cervical cancer have shown limited treatment response rates [6,7]. The patient population is heterogeneous. So, we need easily accessible prognostic factors for the selection of patients for the most appropriate treatment options.

Pre-treatment and post-treatment blood-based SIMs (i.e., NLR, PLR, SII, and SIRI) have been evaluated for prognostic value and even predictive significance in our retrospective study. Most of the studies in the literature are focused on the prognostic significance of some of these parameters at baseline and the early stage of the disease [16,17,18]. Therefore, our study population relatively differs from the literature in terms of the evaluation of “metastatic” cervical cancer patients, all of whom had a line “platinum-taxane-bevacizumab” regimen. They were evaluated according to both “pre-treatment” and “post-treatment” blood-based SIMs, as mentioned before.

Al Jarroudi et al. reported higher baseline NLR and PLR levels as poor prognostic factors for both PFS and OS in inflammatory breast cancer [13]. However, we failed to show any independent prognostic significance of any pre-treatment SIMs, including NLR or PLR, in metastatic CC. However, our post-treatment analysis showed that SIRI was an independent risk factor for OS, while SIRI, SII, and PLR were independent risk factors for PFS, as well.

The studies evaluating SII and SIRI have conflicting outcomes for their prognostic value in the literature. Yeh P et al. demonstrated that pre-hemoglobin, pre-SII, post-SII, and post-SIRI were significant prognostic factors for advanced-stage oropharyngeal cancer, while pre-SII was reported to have no prognostic role in resected pancreas cancer patients [15,21]. In our study, we found out that “post-treatment” SIRI was a significant prognostic factor for OS, whereas “post-treatment” SIRI, SII, and PLR were independent risk factors for PFS, as well. So, post-SIRI stays a step ahead among all SIMs in metastatic cervical cancer. We consider that SIRI differs from others by including monocyte count (i.e., monocyte × neutrophil/lymphocyte counts). Here, “post-treatment” blood samples indicate the levels after progression on bevacizumab-containing treatment. Progression on an antiangiogenic drug might have contributed to a more complex and heterogeneous tumor microenvironment. The tumor microenvironment includes many inflammatory cells, and they spread to the bloodstream in metastatic ones. All of these factors may have a global effect on progression and mortality. So, a monocyte-based SIM, such as post-treatment SIRI, may indicate a more complex and inflamed tumor. Therefore, post-treatment SIRI level can be used as a prognostic factor in metastatic cervical cancer. Additionally, post-SII, but not pre-SII, was shown to be an independent prognostic factor in pancreatic ductal adenocarcinoma patients receiving neoadjuvant therapy [21]. It was correlated with our outcomes, in terms of the significant prognostic role of post-SII, not pre-SII, for PFS (*p* = 0.022) (Table 4).

We also evaluated our patients according to the metastatic status at diagnosis (i.e., metachronous metastasis vs. “de novo” metastasis). The patients with “de novo” metastasis had higher mortality irrespective of any risk factor (*p* < 0.001). The prognostic value of blood-based SIMs, in terms of prognostic significance of “post-treatment” SIRI for OS and “post-treatment” SIRI, SII, and PLR for PFS, was more prominent in the whole population and metachronous metastatic ones. However, they did not differ for “de novo” metastatic ones. The correlated outcomes for the overall population and metachronous metastatic ones might be related to the higher rate of patients with metachronous metastasis in the whole population, dominating the outcomes of the study. Two-thirds (69.2%, n: 92) of the patients had metachronous metastasis. There may be other possible reasons for poor prognosis in the metachronous metastatic subgroup. First of all, progression after definitive locoregional treatment might have led to more resistant clones than “de novo” metastatic ones. These resistant cancer cells might have become more resistant after failure with the first-line chemotherapy and bevacizumab combination. The more resistant tumor cells, the more inflammatory cells in the tumor stromal microenvironment. Therefore, metachronous metastatic patients with higher levels of post-treatment SIMs might have had poor survival outcomes. Second, blood-based SIMs are dynamic markers reflecting the progression of the metastatic process. More inflammatory cells in the tumor microenvironment and bloodstream may mirror lower elimination of tumor cells with systemic treatment. We consider that all of these possible mechanisms might have contributed to shorter survival in “de novo” metastatic cervical cancer patients.

All of our patients had first-line platin–taxane–bevacizumab combination treatment, as mentioned previously. Treatment resistance is a major problem for these patients. It is well-known that viral etiology, such as HPV, has a key role in carcinogenesis. Therefore, it is rational to use immunotherapy in cervical cancer. In the KEYNOTE-826 trial, pembrolizumab addition to chemotherapy w/o bevacizumab demonstrated significant improvements in both PFS and OS in metastatic patients [22]. Nivolumab is another treatment option as a subsequent line [23]. Though immunotherapy is recommended as an effective therapeutic approach in clinical guidelines, it is not reimbursed in many countries, as if in Türkiye. The reimbursement issue makes it a little bit unfeasible. In our study, only 3 (0.2%) of 133 patients had pembrolizumab. Unfortunately, we could not have evaluated the prognostic significance of pre-treatment or post-treatment SIMs in these patients due to the small number of patients. There is limited data for the prognostic role of blood-based SIMs in the literature. High post-NLR and post-SIRI were reported to have prognostic value in head and neck cancer and non-small cell lung cancer [24,25].

There was objective tumor regression with the platin–taxane–bevacizumab triplet regimen. Most of the patients had stable disease with second-line treatment (Table 5). Our analysis highlighted the efficacy of second-line single-agent treatments, and the results were parallel with the literature. One of the notable findings in our study is the improvement in response rates with the addition of bevacizumab to these treatments.

When bevacizumab was not added to the carboplatin–taxane combination, DCR was reduced by half, and no objective response was achieved (Table 5). Similarly, while DCR was 33.3% with gemcitabine–platinum therapy, it increased up to 50% with the addition of bevacizumab. These findings highlight the contribution of bevacizumab to treatment efficacy in our study. “Within the study cohort, only three patients received pembrolizumab, with a DCR of 66.6%. A limited number of patients underwent other treatment modalities.”

For patients considered “platinum-sensitive” after progression on first-line therapy, combination treatment strategies including antiangiogenic therapies appear to be appropriate treatment options with favorable outcomes. Combination of chemotherapeutics and bevacizumab or immunotherapy alone may be considered as good options for others with platin-resistant disease.

There are some limitations in our study. These are its retrospective nature and the relatively limited number of patients. A notable limitation of the study is the potential impact of the heterogeneity in second-line treatment regimens on overall survival outcomes, which may have introduced variability that could not be fully controlled for in the analysis. However, the multicenter design increases the power of the study but also introduces heterogeneity, especially in the second-line treatment choices. Another limitation of our study is the lack of available PD-L1 expression data for the patients, which limited the ability to perform further correlations with tumor microenvironment features.

## 4. Conclusions

In conclusion, there is no well-established standard of care as a second-line setting for advanced-stage cervical cancer patients who have progressed on first-line bevacizumab-containing chemotherapy regimens. Our results indicate that a platinum-based rechallenge strategy, especially including bevacizumab, should be considered as a second-line treatment option for platinum-sensitive CC patients, similar to ovarian cancer, where the interval is longer than six months. Regardless of whether the regimen was single-agent or combination therapy, the addition of anti-angiogenic agents improved response rates and disease control rates, demonstrating that this approach could be a beneficial option. In our study, post-SIRI is determined as an independent risk factor for OS and PFS. High post-SIRI is associated with poorer survival outcomes, possibly due to its inclusion of monocytes, which may reflect the presence of myeloid-derived suppressor cells (MDSCs). Given the immunosuppressive role of MDSCs in the tumor microenvironment, SIRI may more accurately capture the balance of immune activation and suppression than other indices, thereby explaining its stronger prognostic value. High post-SIRI, -SII, and -PLR were also found to be independent risk factors for PFS. The relationship between these SIMs and clinical outcomes was more distinct in the metachronous metastatic group. However, we need randomized clinical trials in this area.

## Figures and Tables

**Table 1:** Baseline characteristics of patients.

Patients’ Characteristics	
Histology n (%)	
SCC	105 (78.9%)
Adenocarcinoma	28 (21.1%)
Age (median(min–max))	51 (min–max: 24–75)
SIRI (median)	
Pretreatment SIRI (pre-SIRI)	2.4
Post-treatment SIRI (post-SIRI)	2.1
NLR (median)	
Pretreatment NLR (pre-NLR)	3.56
Post-treatment NLR (post-NLR)	3.1
SII (median)	
Pretreatment SII (pre-SII)	909
Post-treatment SII (post-SII)	746
PLR (median)	
Pretreatment PLR (pre-PLR)	224
Post-treatment PLR (post-PLR)	197
Stage	
Stage 2	48 (36.1% )
Stage 3	42 (31.6%)
Stage 4	43 (32.3%)

NLR: neutrophil–lymphocyte ratio. PLR: platelet–lymphocyte ratio. SII: Systemic Inflammatory Index. SIRI: System Inflammation Response Index.

**Table 2:** Univariate and multivariate Cox regression analyses for overall survival according to pretreatment factors.

Pretreatment Analysis	Overall Survival					
	Univariate Analysis			Multivariate Analysis		
	HR	CI %95	*p* Value	HR	CI %95	*p* Value
Age <65 vs. >65	1.246	0.793–1.958	0.341			
Histology SCC Adenocarcinoma	1.029	0.601–1.763	0.549			
SIRI (median) High vs. low	1.9	1.192–3.04	**0.007**	1.713	0.95–3.086	0.073
NLR (median) High vs. low	1.639	1.031–2.604	**0.037**	1.191	0.665–2.132	0.557
SII (median) High vs. low	1.554	0.979–2.469	0.61			
PLR (median) High vs. low	0.97	0.612–1.539	0.89			

Values in bold indicate statistical significance (*p* < 0.05).

**Table 3:** Univariate and multivariate Cox regression analyses for overall survival according to post-treatment factors.

Post-Treatment Analysis	Overall Survival					
	Univariate Analysis			Multivariate Analysis		
	HR	CI %95	*p* Value	HR	CI %95	*p* Value
Age <65 vs. >65	1.246	0.793–1.958	0.341			
Histology SCC Adenocarcinoma	1.029	0.601–1.763	0.917			
Post-SIRI High vs. low	2.66	1.639–4.344	**<0.001**	2.11	1.156–3.85	**0.015**
Post-NLR High vs. low	2.5	1.54–4.05	**<0.01**			
Post-SII High vs. low	2.19	1.367–3.533	**0.001**			
Post-PLR High vs. low	1.89	1.195–3.018	**0.07**			

Values in bold indicate statistical significance (*p* < 0.05).

**Table 4:** Univariate and multivariate Cox regression analyses for PFS according to post-treatment factors.

Post-Treatment Analysis	PFS					
	Univariate Analysis			Multivariate Analysis		
	HR	CI %95	*p* Value	HR	CI %95	*p* Value
Age <65 vs. >65	1.247	0.862–1.802	0.241			
Histology SCC Adenocarcinoma	1.316	0.849–2.041	0.22			
Post-SIRI High vs. low	2.409	1.629–3.562	**<0.001**	2.743	1.647–4.567	**<0.001**
Post-NLR High vs. low	1.554	1.060–2.251	**0.024**			
Post-SII High vs. low	1.854	1.266–2.714	**0.002**	0.483	0.259–0.902	**0.022**
Post-PLR High vs. low	1.771	1.214–2.584	**0.003**	1.876	1.144–3.078	**0.013**

Values in bold indicate statistical significance (*p* < 0.05).

**Table 5:** Second-line treatment outcomes.

	n (%)	ORR (%)	DCR (%)
Gemcitabine	25 (29.8)	0	24
Carboplatin/Paclitaxel	9 (10.7)	0	11.1
Carboplatin/ Paclitaxel/ Bevacizumab	9 (10.7)	11.1	22.2
Topotecan	7 (8.3)	0	14.2
Cisplatin/Paclitaxel/Bevacizumab	6 (7.1)	16.6	33.3
Gemcitabine/Cisplatin	3 (3.6)	0	33.3
Topotecan/Bevacizumab	3 (3.6)	0	33.3
Carboplatin/Docetaxel/Bevacizumab	2 (2.4)	0	0
Pembrolizumab	3 (3.6)	0	66.6
Gemcitabine/Cisplatin/Bevacizumab	2 (2.4)	0	50
Docetaxel	2 (2.4)	0	0
Docetaxel/Bevacizumab	1 (1.2)	0	0
Other Regimes	12 (14.3)		

## Data Availability

Due to patient rights and confidentiality regulations in our country, data sharing cannot be conducted directly, but upon request, data can be sent after consultation with the authors and the ethics committee.
